# Supplementary material for: E2F6 initiates stable epigenetic silencing of germline genes during embryonic development
Source: Nat Commun. 2021 Jun 11;12:3582. doi: 10.1038/s41467-021-23596-w (PMC8195999; doi:10.1038/s41467-021-23596-w)
Supplement: Supplementary file 1 — Supplementary Information [file 41467_2021_23596_MOESM1_ESM.pdf]

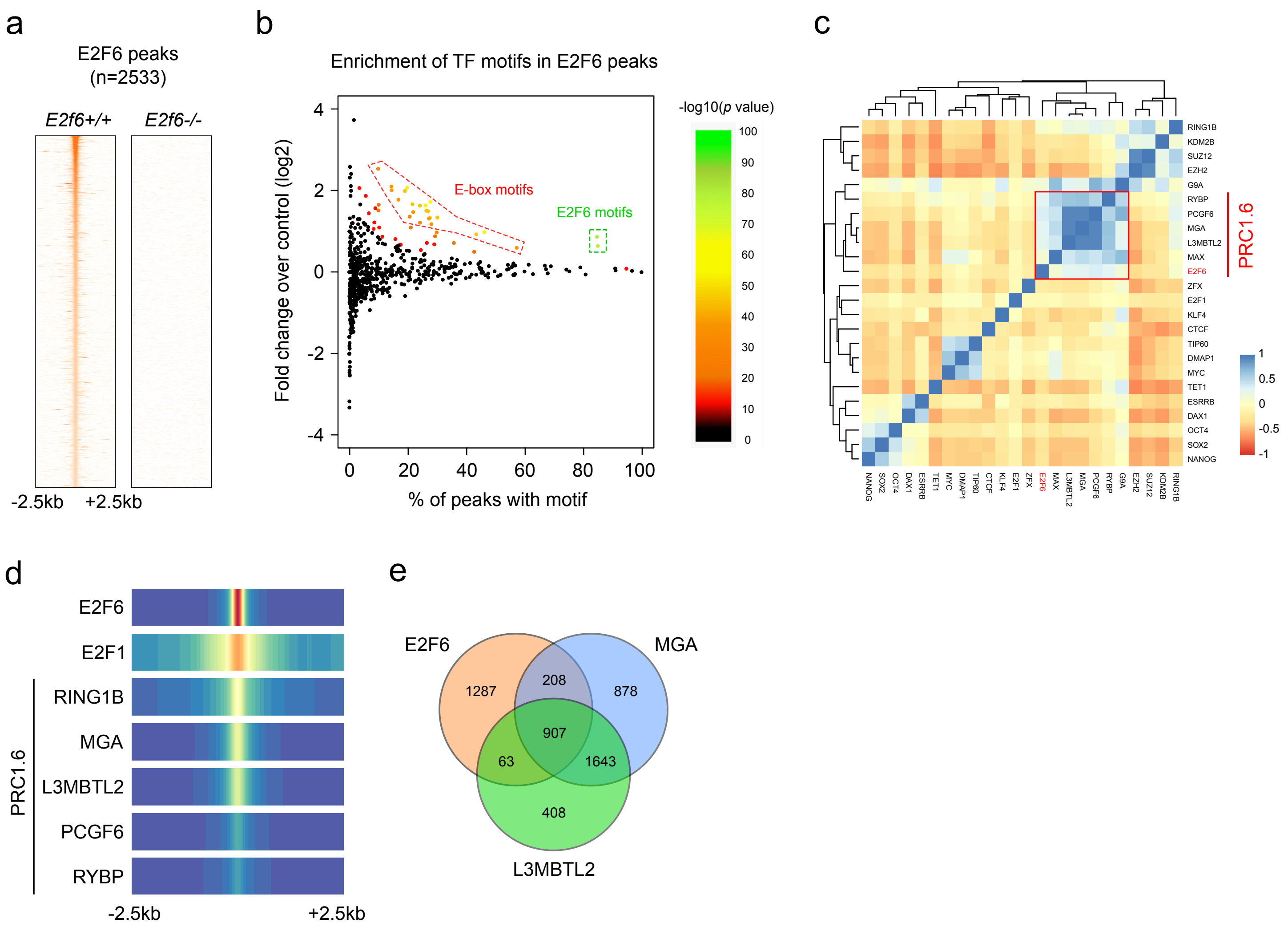

Supplementary Figure 1. Analysis of E2F6 ChIP-seq profiles in mouse ES cells. a. Heatmaps of E2F6 ChIP-seq read densities in *E2f6+/+* and *E2f6-/-* ES cells centered on E2F6 peak summits, demonstrating the specificity of the E2F6 ChIP-seq dataset. **b.** Analysis of JASPAR TF motifs enriched in E2F6 peaks compared to control regions. The color scale indicates the p-values. The most significantly enriched motifs are E2F6 and E-box motifs. **c.** Clustered pairwise correlation matrix between E2F6 ChIP-seq and various ChIP-seq datasets in mouse ES cells. Each point represents the pairwise spearman correlation of normalized read counts in the unified set of peaks from both datasets. The E2F6 dataset clusters with ChIP-seq from other PRC1.6 components (MAX, MGA, L3MBTL2, PCGF6, RYBP) (highlighted in the red square). **d.** Heatmaps of E2F6, E2F1 and PRC1.6 ChIP-seq read densities centered on E2F6 peak summits (plus or minus 2.5 kb). **e.** Venn Diagramm showing the overlap between E2F6, MGA and L3MBTL2 ChIP-seq peaks in mouse ES cells.

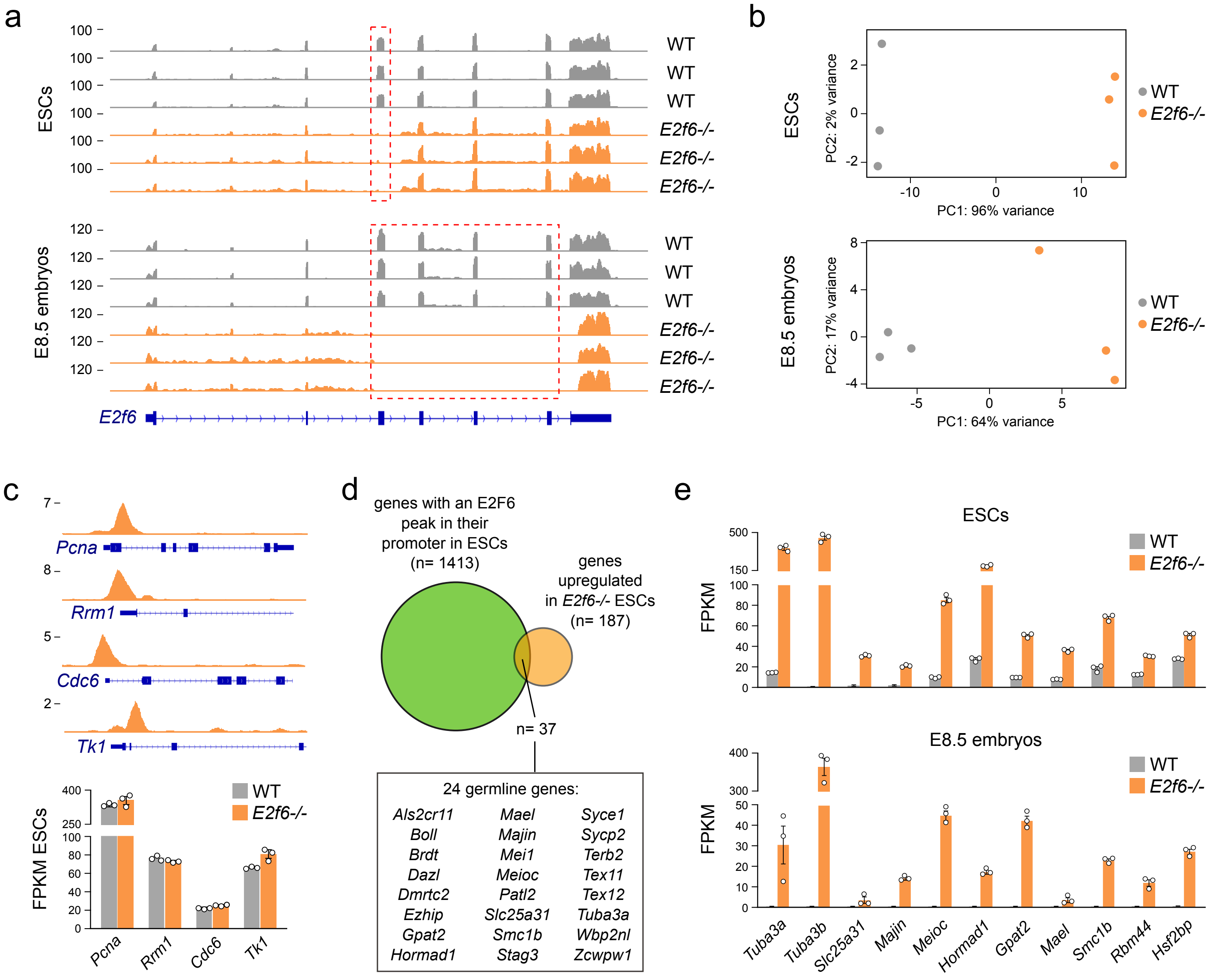

Supplementary Figure 2. Transcriptome analysis in *E2f6-/-* ESCs and E8.5 embryos. a. RNA-seq tracks validate the inactivation of the *E2f6* gene in *E2f6-/-* ESCs and embryos. The missing exons are highlighted by the red rectangles. **b.** Principal component analysis (PCA) of RNA-seq data in ESCs and embryos. **c**. ChIP-seq of E2F6 in the promoters of G1/S genes (top) and expression of G1/S genes (FPKM) in WT and *E2f6-/-* ESCs (bottom, mean ± SEM, n= 3 independent experiments). **d**. Venn diagram comparing the genes with an E2F6 peak in their promoter and the genes upregulated in *E2f6-/-* ES cells. The names of germline genes bound and repressed by E2F6 in ES cells are indicated. **e**. Expression of germline genes (FPKM) in WT and *E2f6-/-* ESCs and embryos (mean ± SEM, n=3 independent experiments for ESCs, n=3 embryos).

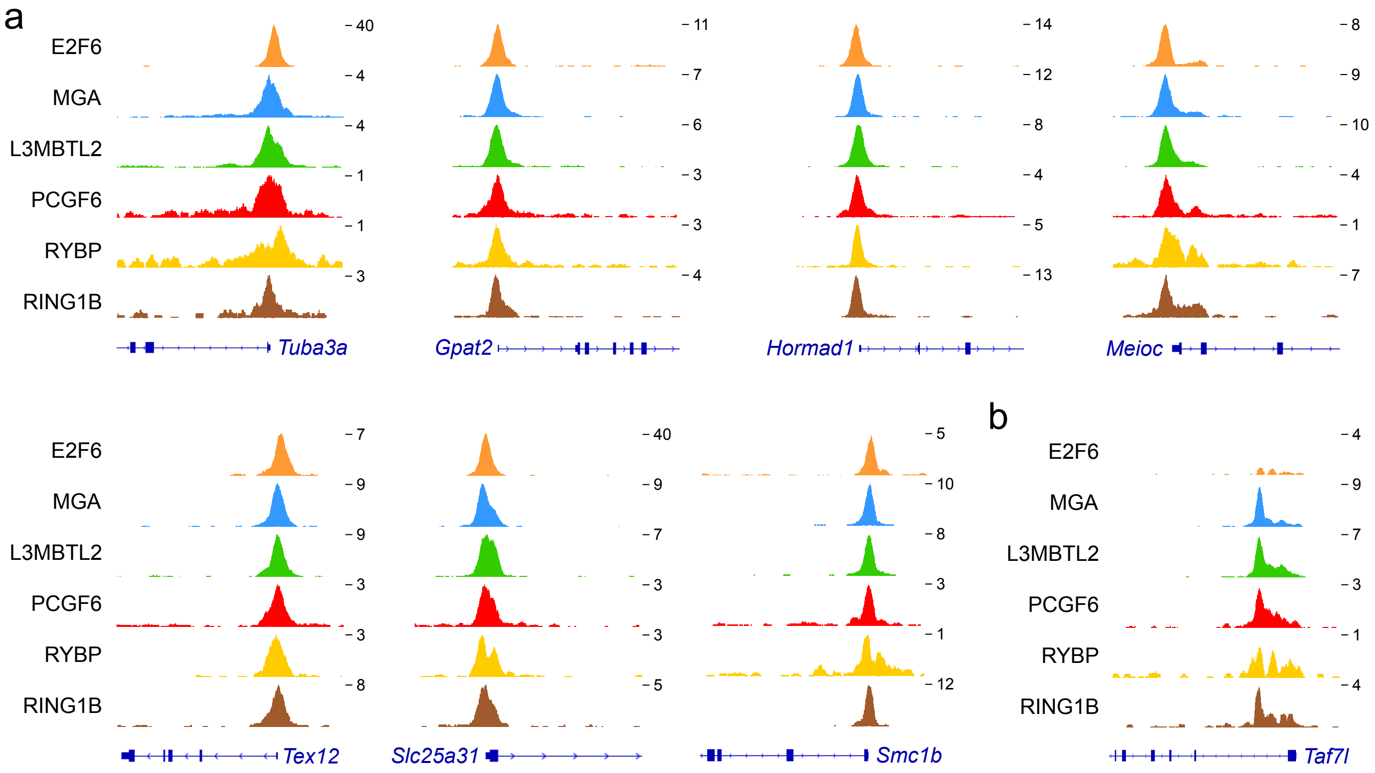

Supplementary Figure 3. ChIP-seq profiles of E2F6 and PRC1.6 at germline genes in mouse ES cells. The figure shows ChIP-seq signals for E2F6 and other PCR1.6 components in mouse ES cells around the promoters of germline genes repressed by E2F6 (**a**). An example of germline gene bound and repressed by MGA alone but not E2F6 is shown (**b**). RefSeq gene annotations are shown below the tracks.

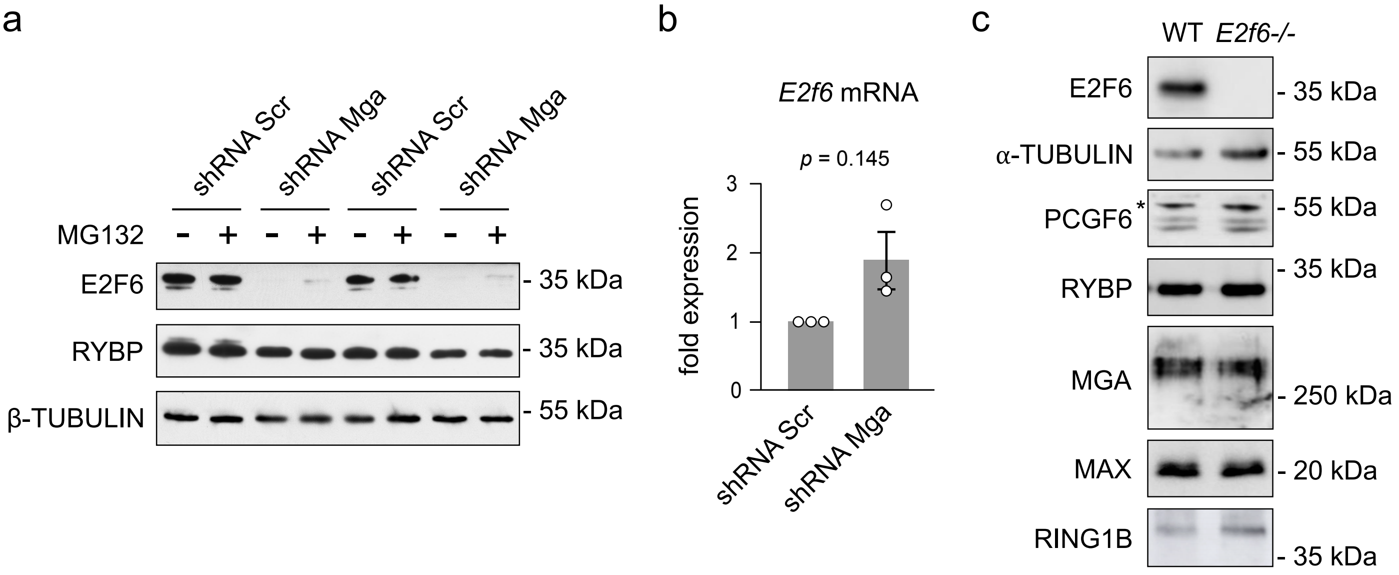

Supplementary Figure 4. MGA depletion causes posttranscriptional downregulation of the E2F6 protein in ES cells. a. Western blot showing reduced levels of E2F6 proteins in *Mga* knockdown ES cells. ES cells were transduced with lentiviral vectors expressing an shRNA against MGA (shRNA Mga) or a scramble control (shRNA scr). After 3 days, half of the cells were treated for 2h with 5μM MG132 to block the proteolityc activity of the proteasome complex. Western blot was performed on whole cell extracts. β-TUBULIN was used as a loading control. **b.** RT-qPCR analysis of *E2f6* mRNA transcript levels in ES cells transduced with an shRNA targeting MGA (shRNA Mga) or a control scramble shRNA (shRNA Scr). The expression levels were normalized to *Actb* and are represented as a fold change relative to shRNA Scr cells (mean ± SEM, n= 3 independent experiments). *p* value: two-tailed t-test. **c.** Western blot of PRC1.6 subunits in *E2f6-/-* compared to WT ES cells. α-TUBULIN was used as a loading control. * non-specific band.

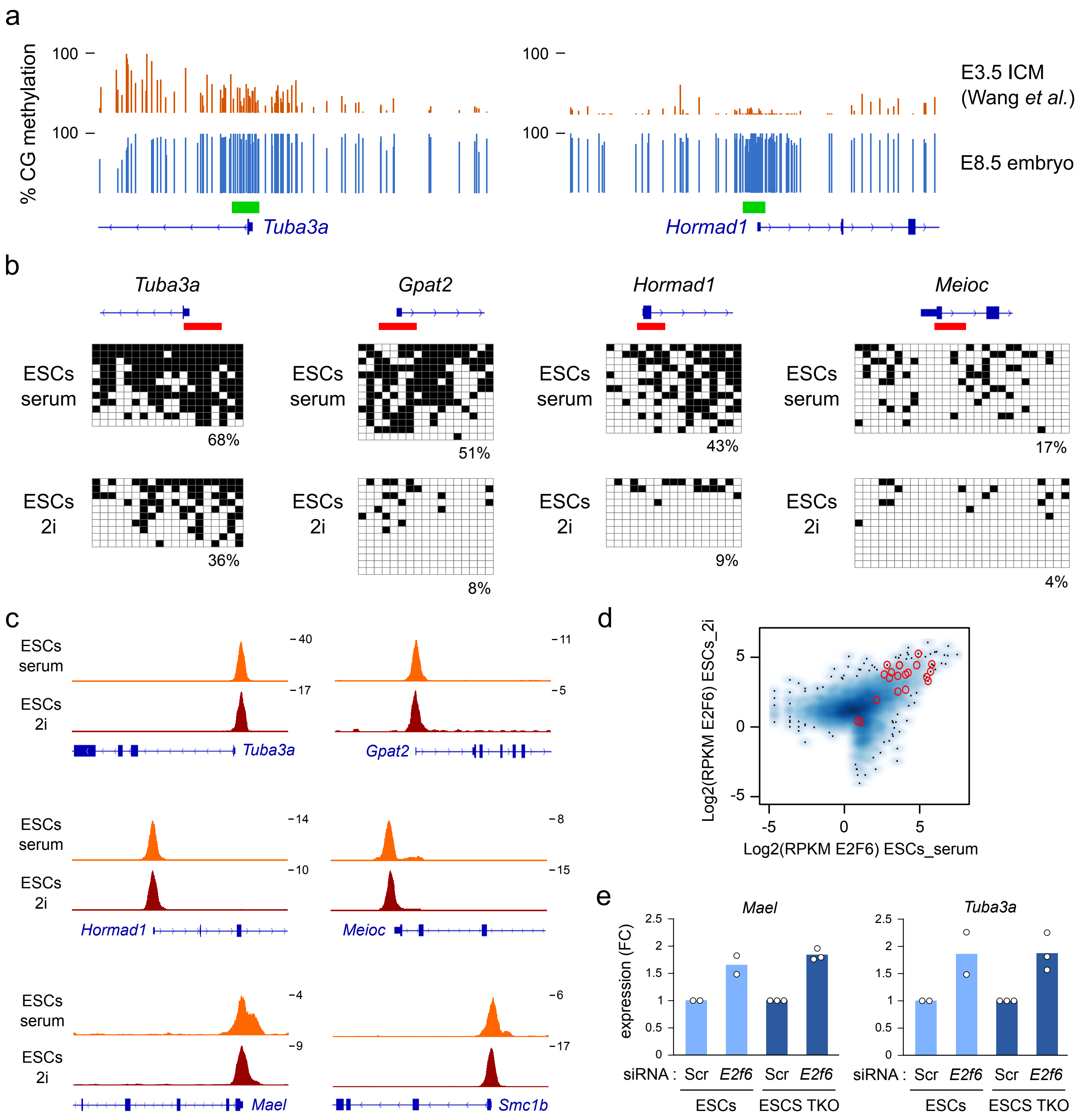

Supplementary Figure 5. Repression of germline genes by E2F6 in hypomethylated embryonic cells. a. WGBS methylation profiles in the promoters of germline genes *Tuba3a* and *Hormad1* in Inner Cell Mass (ICM) (Wang *et al.*, Cell 2014) and E8.5 embryos. Green boxes represent CpG islands. **b.** Bisulfite sequencing of germline gene promoters in ESCs cultured in serum or 2i conditions. For each gene the red line represents the position of the bisulfite amplicon and the squares represent the methylation of CpG sites (white: unmethylated, black: methylated) in individually sequenced clones. **c.** E2F6 ChIP-seq binding profiles at germline genes in ESCs cultured in serum or 2i conditions (Tao *et al.*, Stem Cell Reports 2020). **d.** Global correlation of E2F6 ChIP-seq read density in peak regions between serum and 2i ESCs. Red points represent peaks in germline gene promoters repressed by E2F6. **e.** RT-qPCR analysis of *Mael* and *Tuba3a* expression following siRNA knockdown of *E2f6* in WT and *Dnmt*-TKO ES cells. Expression is represented as a fold change (FC) relative to the scrambled siRNA (Scr) control (expression normalized to *B2m*, *Gusb* and *Rpl13a*, n=2 independent replicates for ESCs, n=3 independent replicates for ESCs TKO).

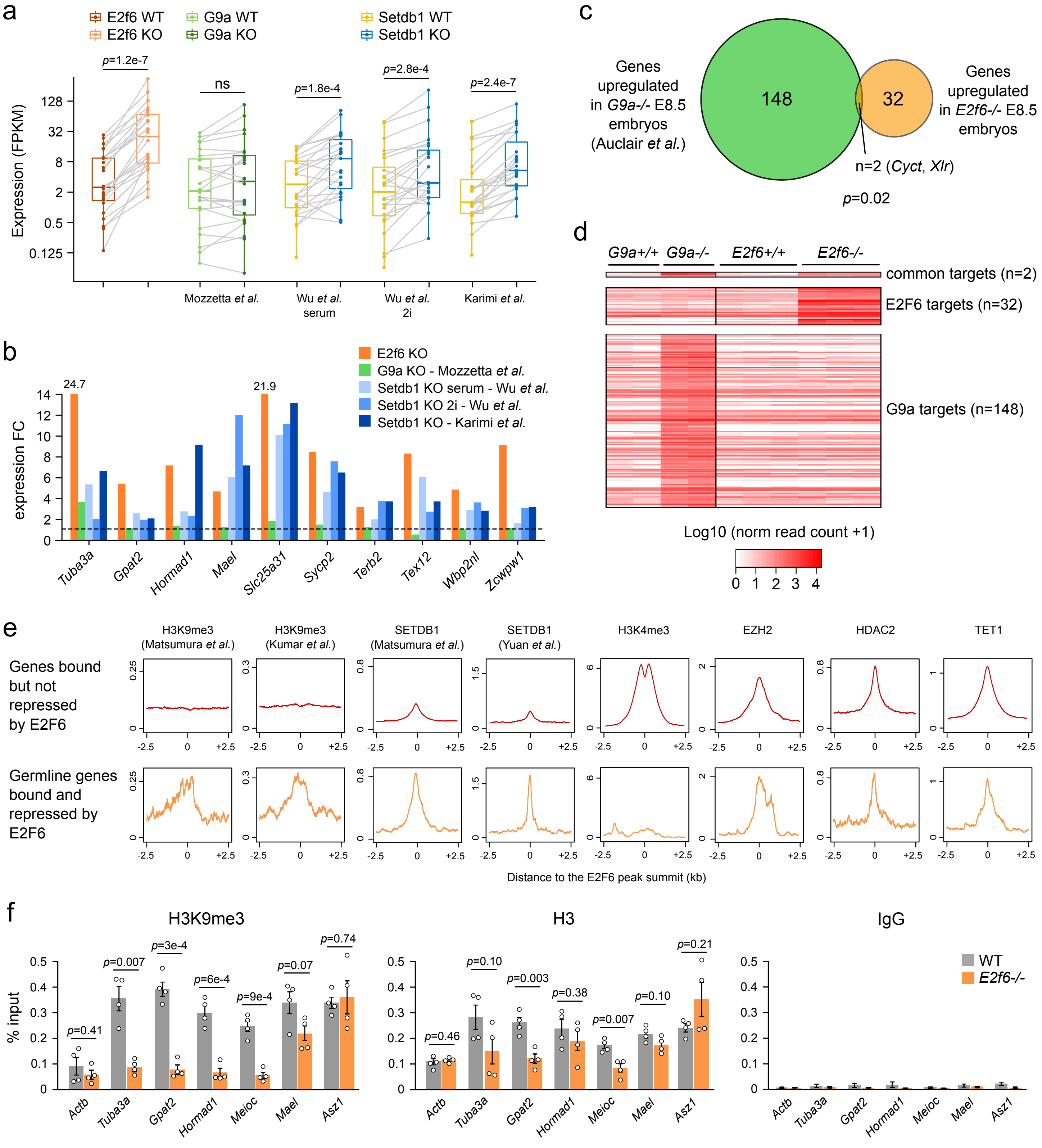

Supplementary Figure 6. E2F6 stimulates the deposition of H3K9me3 in the promoters of germline genes in naïve ES cells. a. Boxplots showing the expression of germline genes repressed by E2F6 in ESCs (n=24 genes) in *E2f6* KO, *G9a* KO (Mozzetta *et al.*, Mol Cell 2014) and SETDB1 KO (Wu *et al.*, Cell Rep 2020, Karimi *et al.*, Cell Stem Cell 2011) ESCs. *p* values: paired two sided Wilcoxon test. ns: not significant. In the boxplots the line indicates the median, the box limits indicate the upper and lower quartiles and the whiskers extend to the limits of the distribution. **b.** Expression of selected germline genes in *E2f6* KO, *G9a* KO and *Setdb1* KO ESCs (fold change expression calculated by DESeq2 in KO vs WT ESCs). **c.** Venn Diagram comparing the genes upregulated in *E2f6-/-* and *G9a-/-* E8.5 embryos (Auclair *et al.*, Genome Res 2016). *p*-value: hypergeometric test. **d.** Heatmap of the expression of E2F6 and G9a target genes (normalized read count + 1) in *E2f6* KO and *G9a* KO embryos. Each column is an individual embryo. **e.** Metaplots of ChIP-seq signals in germline gene promoters repressed by E2F6 (n=24) compared to gene promoters bound but not repressed by E2F6 (n=1378). **f.** ChIP-qPCR quantification of H3K9me3, H3 and IgG control in the promoters of E2F6 target genes in *E2f6+/+* and *E2f6-/-* ES cultured in 2i conditions (mean ± SEM, n=4 independent experiments). *Actb* was used as a negative control and *Asz1* is a germline gene not repressed by E2F6. *p*-values: two-tailed t-test.

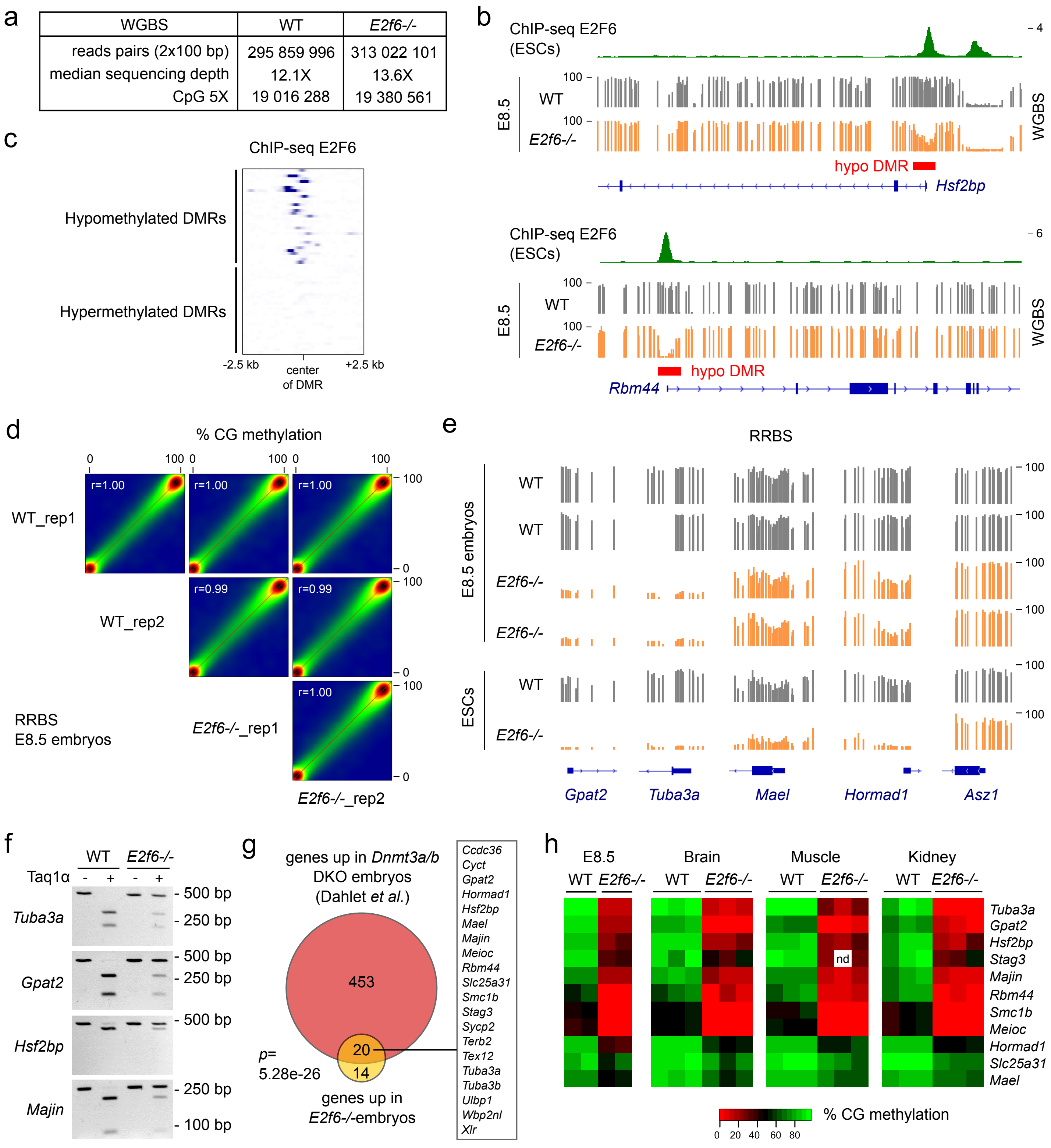

WGBS	WT	*E2f6-/-*
reads pairs (2x100 bp)	295 859 996	313 022 101
median sequencing depth	12.1X	13.6X
CpG 5X	19 016 288	19 380 561

Supplementary Figure 7. DNA methylation analysis in *E2f6-/-* mice and ES cells. a. Sequencing information for the WGBS libraries in WT and *E2f6-/-* E8.5 embryos. **b.** Genome tracks of E2F6 ChIP-seq (ES cells) and WGBS in WT and *E2f6-/-* E8.5 embryos over the *Hsf2bp* and *Rbm44* genes. Hypomethylated DMRs (hypo DMRs) are depicted by red rectangles. Refseq gene annotations are shown below the tracks. **c.** Heatmap of E2F6 ChIP-seq signals in 2.5 kb around the center of hypomethylated and hypermethylated DMRs. Hypo DMRs frequently colocalize with E2F6 peaks. **d.** Density scatter plots showing the correlation of methylation scores in 500 bp windows between RRBS replicates in E8.5 embryos. The Pearson correlation coefficients are indicated in the top left corner. **e.** Examples of RRBS promoter methylation profiles for E2F6 target genes in WT and *E2f6-/-* E8.5 embryos (top) and ESCs (bottom). A germline gene not repressed by E2F6 (*Asz1*) is shown as a control. **f.** DNA methylation analysis by COBRA in the promoters of E2F6 target genes in WT and *E2f6-/-* E8.5 embryos. **g.** Venn diagram comparing the genes upregulated in *E2f6-/-* and *Dnmt3a/b* DKO E8.5 embryos (Dahlet *et al.*, Nature Comm 2020). *p*-value: hypergeometric test. **h.** Heatmaps of promoter CpG methylation of E2F6 target genes profiled by RRBS in organs from WT and *E2f6-/-* mice (4 weeks of age). Each column represents an independent animal. The values in E8.5 embryos are shown for comparison. nd: not determined.

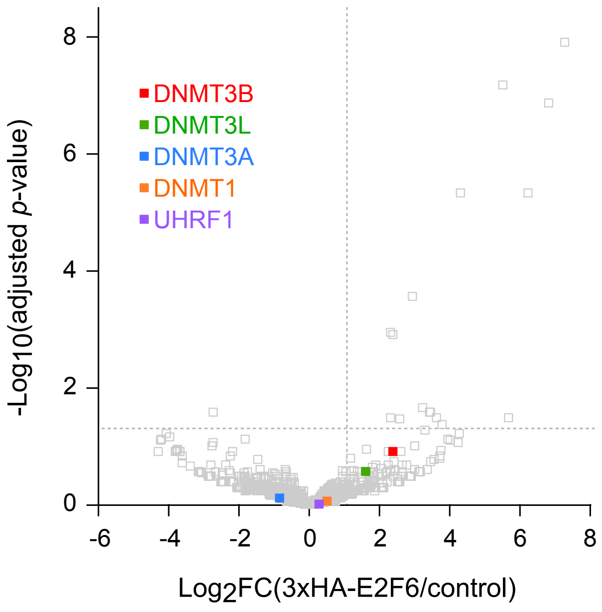

Supplementary Figure 8. Interaction between E2F6 and the DNA methylation machinery investigated by mass spectrometry in ES cells. The Volcano plot shows the position of DNMTs and UHRF1 (colored dots) in the E2F6 interactome identified by mass-spectrometry in ES cells expressing 3xHA-E2F6.

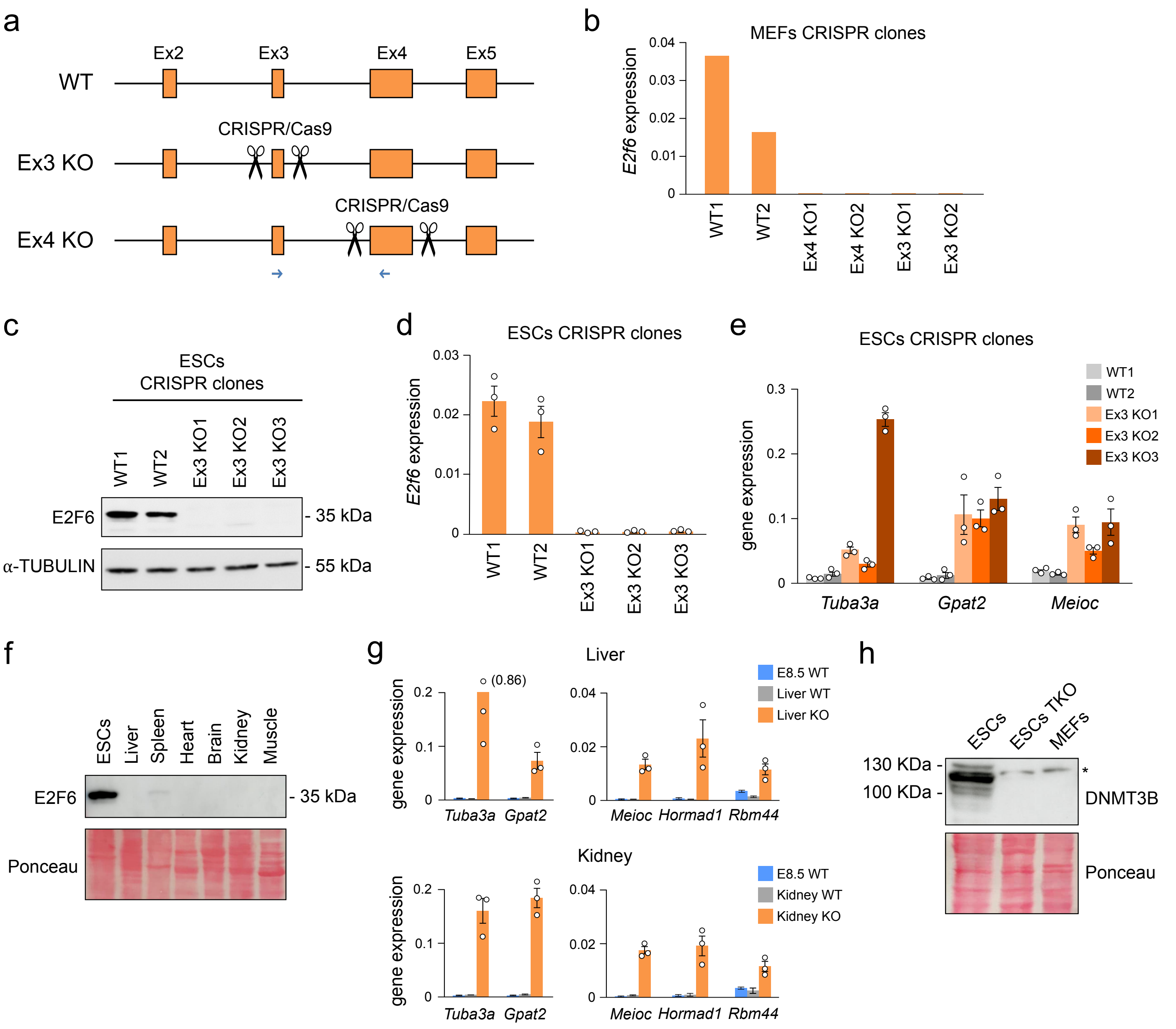

Supplementary Figure 9. Validation of *E2f6* CRISPR-Cas9 clones and expression of E2F6 in postnatal organs. a. Strategy used for inactivation of the *E2f6* gene by CRISPR-Cas9. gRNAs were designed on each side of exons to remove either the exon 3 or the exon 4 and create a frameshit to prevent expression of the E2F6 protein. Blue arrows depict the oligos used for validation of the exon deletion by RT-qPCR. **b.** RT-qPCR analysis of *E2f6* expression in MEFs CRISPR-Cas9 clones using oligos spanning the exon 3-4 region (n=1 replicate per clone). **c.** Western blot of E2F6 in ESCs CRISPR-Cas9 clones. α-TUBULIN was used as a loading control. **d.** RT-qPCR analysis of *E2f6* expression in ESCs CRISPR-Cas9 clones using oligos spanning the exon 3-4 region (n=3 replicates per clone). **e.** RT-qPCR analysis of the expression of three E2F6 target genes in ESCs WT and *E2f6*-KO CRISPR-Cas9 clones (mean ± SEM, n=3 replicates per clone). **f.** Western blot showing the abundance of the E2F6 protein in mouse organs at 4 weeks of age compared to ES cells. **g.** RT-qPCR analysis of the expression of E2F6 target genes in WT and *E2f6* KO liver and kidney at 4 weeks of age, as well as WT E8.5 embryos for comparision (expression normalized to *Actb* and *Rpl13a*, mean ± SEM, n=2 embryos, n=3 organs). **h.** Western blot of DNMT3B in WT ES cells, *Dnmt* TKO ES cells and MEFs. *: unspecific band.
