# Supplementary material for: E2F6 initiates stable epigenetic silencing of germline genes during embryonic development
Source: Nat Commun. 2021 Jun 11;12:3582. doi: 10.1038/s41467-021-23596-w (PMC8195999; doi:10.1038/s41467-021-23596-w)
Supplement: Supplementary file 10 — Description of Additional Supplementary Files [file 41467_2021_23596_MOESM10_ESM.docx]

Description of additional supplementary information

Title: Supplementary Data 1

Description: Sequencing statistics.

Title: Supplementary Data 2

Description: E2F6 ChIP-seq peaks.

Title: Supplementary Data 3

Description: E2F6 interactome.

Title: Supplementary Data 4

Description: Gene expression in E2f6KO ESCs.

Title: Supplementary Data 5

Description: Gene expression in E2f6KO embryos.

Title: Supplementary Data 6

Description: WGBS DMRs.

Title: Supplementary Data 7

Description: Primer sequences.
