# Supplementary material for: E2F6 initiates stable epigenetic silencing of germline genes during embryonic development
Source: Nat Commun. 2021 Jun 11;12:3582. doi: 10.1038/s41467-021-23596-w (PMC8195999; doi:10.1038/s41467-021-23596-w)
Supplement: Supplementary file 11 — Reporting Summary [file 41467_2021_23596_MOESM11_ESM.pdf]

nature research

Corresponding author(s): Michael WEBER

Last updated by author(s): Apr 26, 2021

Reporting Summary

Nature Research wishes to improve the reproducibility of the work that we publish. This form provides structure for consistency and transparency in reporting. For further information on Nature Research policies, see our Editorial Policies and the Editorial Policy Checklist.

Statistics

For all statistical analyses, confirm that the following items are present in the figure legend, table legend, main text, or Methods section.

n/a	Confirmed	
☐	☒	The exact sample size (*n*) for each experimental group/condition, given as a discrete number and unit of measurement
☐	☒	A statement on whether measurements were taken from distinct samples or whether the same sample was measured repeatedly
☐	☒	The statistical test(s) used AND whether they are one- or two-sided<br>*Only common tests should be described solely by name; describe more complex techniques in the Methods section.*
☒	☐	A description of all covariates tested
☒	☐	A description of any assumptions or corrections, such as tests of normality and adjustment for multiple comparisons
☐	☒	A full description of the statistical parameters including central tendency (e.g. means) or other basic estimates (e.g. regression coefficient) AND variation (e.g. standard deviation) or associated estimates of uncertainty (e.g. confidence intervals)
☐	☒	For null hypothesis testing, the test statistic (e.g. *F*, *t*, *r*) with confidence intervals, effect sizes, degrees of freedom and *P* value noted<br>*Give P values as exact values whenever suitable.*
☒	☐	For Bayesian analysis, information on the choice of priors and Markov chain Monte Carlo settings
☒	☐	For hierarchical and complex designs, identification of the appropriate level for tests and full reporting of outcomes
☐	☒	Estimates of effect sizes (e.g. Cohen's *d*, Pearson's *r*), indicating how they were calculated

Our web collection on statistics for biologists contains articles on many of the points above.

Software and code

Policy information about availability of computer code

Data collection	All software and code used is open source and publicly available. Data was processed using Trim Galore v0.4.2 and v0.4.4, BSMAP v2.74, Bismark v0.18.2, bowtie2 v2.3.0, TopHat v2.0.13, Mascot v2.5.
Data analysis	All software and code used for data analysis is open source and publicly available. Data analysis was performed using the R statistical software v3.3.2 and v3.5.0, HTSeq v0.7.2, DESeq2 v1.16.1, DAVID v6.8, R methylKit package v1.6.3, bedtools v2.25.0, Peakzilla, TFmotifView, MAST v5.1.0, bwtool v1.0, R NMF package v0.21.0, R ChIPpeakAnno package v3.18.2, the Integrative Genomics Viewer (IGV) v2.8.3, the BISMA software, EdgeR v3.24.3, Proline v1.4.

For manuscripts utilizing custom algorithms or software that are central to the research but not yet described in published literature, software must be made available to editors and reviewers. We strongly encourage code deposition in a community repository (e.g. GitHub). See the Nature Research guidelines for submitting code & software for further information.

Data

Policy information about availability of data

All manuscripts must include a data availability statement. This statement should provide the following information, where applicable:

- Accession codes, unique identifiers, or web links for publicly available datasets
- A list of figures that have associated raw data
- A description of any restrictions on data availability

The RNA-seq, ChIP-seq, RRBS and WGBS data generated in this study are available in the NCBI Gene Expression Omnibus (GEO) under the accession number GSE149025. The mass spectrometry proteomics data have been deposited to the ProteomeXchange Consortium via the PRIDE partner repository with the dataset identifier PXD023974. We used the following publicly available databases: UCSC genome annotations, Swissprot database. All relevant data are available in the

Supplementary Information files or from the corresponding authors. The Source data for Figs. 1h, j, 2b, c, f, 3c-e, 4b, c, e, f, 5a, b, h, 6b, c, e-g and Supplementary Figs. 2c, e, 4a-c, 5e, 6e, 7f, 9c-h are provided in the Source data file.

Field-specific reporting

Please select the one below that is the best fit for your research. If you are not sure, read the appropriate sections before making your selection.

☒ Life sciences ☐ Behavioural & social sciences ☐ Ecological, evolutionary & environmental sciences

For a reference copy of the document with all sections, see nature.com/documents/nr-reporting-summary-flat.pdf

Life sciences study design

All studies must disclose on these points even when the disclosure is negative.

Sample size	No statistical method was used to predetermine sample size. For statistical treatment, we chose to perform at least 3 independent replicates whenever possible.
Data exclusions	No data were excluded from the analyses.
Replication	All experimental findings were reproduced in independent replicates as described in the figure legends. We performed 5 independent replicates for the interactome study and 3 independent replicates for ChIP-qPCR experiments. RNA-seq experiments were performed in 3 independant samples.
Randomization	The samples were allocated into experimental groups based on the genotype (WT vs E2f6-/-). The cell lines were cultured simultaneously and the animal samples were taken from the same litters to minimize experimental variations.
Blinding	The investigators were not blinded to group allocation during experiments and data analysis because the samples needed to be genotyped before performing the experiments.

Reporting for specific materials, systems and methods

We require information from authors about some types of materials, experimental systems and methods used in many studies. Here, indicate whether each material, system or method listed is relevant to your study. If you are not sure if a list item applies to your research, read the appropriate section before selecting a response.

Materials & experimental systems

n/a	Involved in the study	
☐	☒	Antibodies
☐	☒	Eukaryotic cell lines
☒	☐	Palaeontology and archaeology
☐	☒	Animals and other organisms
☒	☐	Human research participants
☒	☐	Clinical data
☒	☐	Dual use research of concern

Methods

n/a	Involved in the study	
☐	☒	ChIP-seq
☒	☐	Flow cytometry
☒	☐	MRI-based neuroimaging

Antibodies

Antibodies used	E2F6 (LLF6-2, Kerafast)<br>E2F6 (ab53061, Abcam)<br>ACTB (A2066, Sigma Aldrich)<br>□□□□a□-TUBULIN (T9026, Sigma Aldrich)<br>b-TUBULIN (T8328, clone AA2, Sigma Aldrich)<br>RYBP (AB3637, Millipore)<br>L3MBTL2 (39570, Active Motif)<br>PCGF6 (24103-1-AP, Proteintech)<br>MGA (PA5-59934, Thermo Fisher Scientific)<br>RING1B (D139-3, clone 3-3, MBL)<br>MAX (sc-197, Santa Cruz Biotechnology)<br>GLP (PP-B0422-00, clone B0422, R&D Systems)<br>DNMT3B (ab13604, clone 52A1018, Abcam)<br>HA (3F10, Roche)<br>E2F1 (C-20, sc-193, Santa Cruz Biotechnology)<br>H3K9me3 (ab8898, Abcam)<br>H3K4me3 (003-050, Diagenode)

Mouse IgG (12-371, Millipore)
IgG2A (MAB0031, R&D Systems)

Validation

The E2F6 LLF6-2 antibody is validated for western blot in mouse by knock-out experiments in Fig 6 and Supp Fig 4.
The E2F6 ab53061 antibody is validated for ChIP in mouse in Fig 2c and Supp Fig 1a.
The RYBP AB3637 antibody has been validated for western blot in mouse by knock-out experiments in several publications described on the manufacturer's website (ex: PMID 16055728).
The L3MBTL2 39570 antibody has been validated for western blot in mouse by knock-out experiments in PMID 33523934.
The PCGF6 24103-1-AP antibody has been validated for western blot in human cells by knock-out experiments in PMID 29381691.
The RING1B D139-3 antibody has been validated for western blot in mouse by knock-out experiments in several publications (ex: PMID 26526724).
The MAX sc-197 antibody has been validated for western blot in mouse by knock-out experiments in several publications (ex: PMID 29539429).
The GLP PP-B0422-00 antibody has been validated for western blot in mouse by knock-out experiments in several publications (ex: PMID 24389103).
The DNMT3B ab13604 antibody has been validated for western blot in mouse by knock-out experiments in several publications (ex: PMID 33004415) and Supp Fig 9h.
The E2F1 sc-193 antibody has been validated for western blot in mouse and ChIP in mouse in several publications (ex: PMID 26619117).
The ACTB has been validated for western blot in mouse in several publications detailed on the manufacturer's website.
The a-TUBULIN has been validated for western blot in mouse in several publications detailed on the manufacturer's website.
The b-TUBULIN has been validated for western blot in mouse in several publications detailed on the manufacturer's website.
The HA antibodies have been validated for western blot in mouse in several publications detailed on the manufacturer's website.
The H3K9me3 antibody is a ChIP grade antibody validated for ChIP in mouse on the manufacturer's website (https://www.abcam.com/histone-h3-tri-methyl-k9-antibody-chip-grade-ab8898.html).
The H3K4me3 antibody is a ChIP grade antibody validated for ChIP in mouse on the manufacturer's website (https://www.diagenode.com/en/p/h3k4me3-polyclonal-antibody-premium-50-ug-50-ul).

Eukaryotic cell lines

Policy information about cell lines

Cell line source(s)

WT and E2F6-/- ES cells were derived from blastocysts. The E14TG2a ES cell line was obtained from ATCC. J1 WT and J1 Dnmt-TKO ES cell lines were a gift from M. Okano. Mouse embryonic fibroblasts (MEFs) were derived from E13.5 embryos.

Authentication

The cell lines were authenticated by DNA genotyping.

Mycoplasma contamination

All cell lines were tested negative for mycoplasma contamination.

Commonly misidentified lines
(See ICLAC register)

No misidentified cell lines were used in the study.

Animals and other organisms

Policy information about studies involving animals; ARRIVE guidelines recommended for reporting animal research

Laboratory animals

We used an E2f6 knockout mouse line (E2f6<tm1Lees>, PMID: 18366140) maintained on the C57BL/6J background.
Mice (females and males) were euthanasized at E8.5, E13.5 and 4 weeks after birth.

Wild animals

The study did not involve wild animals.

Field-collected samples

The study did not involve samples collected from the field.

Ethics oversight

Animal experimental procedures complied with the ethical regulations of the Comité d'Ethique Régional en Expérimentation Animale de Strasbourg (CREMEAS).

Note that full information on the approval of the study protocol must also be provided in the manuscript.

ChIP-seq

Data deposition

☒ Confirm that both raw and final processed data have been deposited in a public database such as GEO.

☒ Confirm that you have deposited or provided access to graph files (e.g. BED files) for the called peaks.

Data access links
May remain private before publication.

Reviewer token for GEO record GSE149025: glkvicoyvfsjvop.

Files in database submission

ChIP-seq_E2F6_ESCs_WT.fastq
ChIP-seq_input_ESCs_WT.fastq
ChIP-seq_E2F6_ESCs_E2f6KO.fastq
ChIP-seq_input_ESCs_E2f6KO.fastq
ChIP-seq_E2F6_ESCs_WT.bw
ChIP-seq_input_ESCs_WT.bw
ChIP-seq_E2F6_ESCs_E2f6KO.bw
ChIP-seq_input_ESCs_E2f6KO.bw

Genome browser session
(e.g. UCSC)

bw files are provided in the GEO submission for visualization in any genomic browser.

Methodology

Replicates

We sequenced one replicate of ChIP and input samples from WT ES cells, and one replicate of ChIP and input samples from E2f6-/- ES cells.

Sequencing depth

Sequencing was performed in single-end 1x50bp.
ChIP-seq_E2F6_ESCs_WT: 34 671 827 total reads, 24 688 544 uniquely mapped reads
input_ESCs_WT: 45 696 228 total reads, 35 023 164 uniquely mapped reads
ChIP-seq_E2F6_ESCs_E2f6KO: 27 625 633 total reads, 19 647 941 uniquely mapped reads
input_ESCs_E2f6KO: 45 292 901 total reads, 34 308 821 uniquely mapped reads

Antibodies

Anti-E2F6 antibody (ab53061, Abcam)

Peak calling parameters

Reads were cleaned to remove low quality bases and adapters with Trim Galore v0.4.4 using the parameters -q 20 -O 2 -a AGATCGGAAGAGC. Reads were aligned to the mouse mm10 genome using bowtie2 v2.3.0 and only reads mapping with a quality above 10 were kept using samtools. Peaks were called using Peakzilla with default settings.

Data quality

We ensured data quality by performing a saturation analysis of ChIP-seq peaks and verifying that all E2F6 peaks are absent in E2f6-/- ES cells.

Software

Read density tracks were generated using genomeCoverageBed from bedtools from reads extended to 200bp. The analysis of motif enrichment and distribution of motifs in peaks was performed using TFmotifView with motifs from the JASPAR2020 database. Average ChIP-seq signals around E2F6 peak summits were calculated using bwtool v1.0.
